# Up-regulated ENO1 promotes the bladder cancer cell growth and proliferation via regulating β-catenin

**DOI:** 10.1042/BSR20190503

**Published:** 2019-09-09

**Authors:** Mingfei Ji, Zhijun Wang, Jie Chen, Liqiong Gu, Ming Chen, Yelei Ding, Tao Liu

**Affiliations:** 1 Department of Urology, Changzheng Hospital, The Second Military Medical University, Shanghai, China; 2 Department of Ophthalmology, Shanghai East Hospital, Tongji University, Shanghai, China

**Keywords:** β-catenin, apoptosis, bladder cancer, cell cycle, ENO1

## Abstract

Bladder cancer (BC) is the ninth most common malignancy throughout the world. The molecular mechanisms of this disease remain largely unclear. The glycolytic enzyme enolase 1 (ENO1) has been shown to regulate the development of various cancers. However, the significance of ENO1 in BC is underdetermined. In this study, we found that ENO1 was highly expressed in BC tissues and cells. High expression of ENO1 was associated with the poor survival of BC patients. Using lentivirus-mediated knockdown and over-expression, we revealed that ENO1 was critical for the growth and proliferation of BC cells. ENO1 over-expression also promoted the proliferation of SV-HUC-1 cells. At the molecular level, the cell cycle and apoptosis related genes were regulated by ENO1. β-catenin expression was positively regulated by ENO1. Furthermore, ectopic expression of β-catenin reversed the effect of ENO1 knockdown on T24 cell proliferation and growth. Opposite results were observed in β-catenin knockdown T24 cells. Our findings suggested that ENO1 functioned as an oncogene in BC through regulating cell cycle, apoptosis and β-catenin. Targeting ENO1/β-catenin cascade may benefit for BC patients.

## Introduction

Bladder cancer (BC) is the 9th most common and the 13th most lethal malignancy worldwide, with a higher incidence in male than in female [1–3]. More than 80 percent of BC originates from bladder urothelium [4]. Once diagnosed, the prognosis is poor in most of the patients. Endoscopic resection is the routine therapy for BC patients, while its effectiveness remains limited. Recently, increasing evidences have identified some oncogenes and tumor suppressors, including HRAS, FGFR3, TP53 and PTEN that participate in the progression of this disease [5–10]. However, the efficacy of the treatments targeted these genes is far from satisfactory. Therefore, illumination of the molecular events may help us gain insight into the disease progression and potential targets for BC.

Similar to other tumors, BC prefers to catabolize glucose through glycolysis even in enough oxygen condition, a phenomenon known as Warburg effect [11]. The enolase 1 (ENO1) is a glycolytic enzyme that catalyzes the conversion of 2-phosphoglycerate to phosphoenolpyruvate, leading to enhanced glycolysis [12]. Dys-regulation of ENO1 is widely observed in cancer disease. For example, ENO1 is a potential biomarker for hepatocellular carcinoma (HCC) [13]. It serves as a target for LncRNA P5848 and promotes tumor growth and survival in HCC [14]. Targeting ENO1 suppresses the proliferation and growth of gastric cancer cells [15]. In addition, ENO1 is also involved in the development of other cancers, including retinoblastoma [16], glioma [17], pancreatic cancer [18] and lung adenocarcinomas [19]. However, the significance of ENO1 in BC remains to be determined.

β-catenin is an important component of Wnt signaling pathway and plays a key role in regulating cell growth, apoptosis, differentiation and embryonic development [20–22]. When translocating into the nucleus, β-catenin forms complexes with the T-cell factor (TCF)/lymphoid enhancer factor (LEF) family members and becomes active, leading to up-regulation of its target genes, such as c-MYC, cyclin-D1 and epidermal growth factor receptor [23–26]. Thus, ectopic expression or activation of β-catenin promotes the development of various cancers, including BC [27,28]. However, the upstream regulator of β-catenin is largely unknown in BC.

In this study, we aimed to investigate the clinical relevance and the role of ENO1 in BC. We found that ENO1 expression was more abundant in BC tissues and cells. ENO1 over-expression led to increased BC cell proliferation and growth. In contrast, knockdown of ENO1 suppressed the proliferation of BC cells. Mechanistically, cell cycle and apoptosis related genes were regulated by ENO1. ENO1 also served as a stimulator of β-catenin. β-catenin over-expression rescued the suppressed viability in ENO1 silenced BC cells. We speculated that ENO1 promoted BC through up-regulating β-catenin.

## Materials and methods

### Measurement of ENO1 mRNA expression in bladder cancer patients

The primary bladder cancer and normal tissues were obtained from patients of invasive bladder cancer who underwent radical cystectomy at the Changzheng Hospital during 2011–2017. All the patients were newly diagnosed as transitional cell carcinomas by the pathologists and none of them had received systemic treatment. A written informed consent was obtained from each patient. All the procedures were approved by the Ethics Committee of Changzheng Hospital, The Second Military Medical University. The research was carried out in accordance with the World Medical Association Declaration of Helsinki. The median age of the patients was 61 years by the time of surgery. The mRNA expression of ENO1 was determined by quantitative real-time PCR (qRT-PCR) assay.

### ENO1 transcript analysis in bladder cancer patients from TCGA database

ENO1 transcriptome expression datasets were analyzed from websites of The Cancer Genome Atlas (http://cancergenome.nih.gov). A total of 414 bladder cancer tissues and 30 normal tissues were included.

### Correlation between ENO1 expression and BC patients’ survival from TCGA database

The correlation between ENO1 expression (low and high expression) and the survival time of BC patients was analyzed from websites of The Cancer Genome Atlas (http://cancergenome.nih.gov). The bladder cancer patients were divided into ENO1 high expression (*n* = 204) and low expression group (*n* = 205).

### Cell culture

Human bladder epithelial cells SV-HUC-1 and bladder cancer cells T24, 5637, J28, SW780 and RT4 were obtained from ATCC. All the cells were cultured in Dulbecco modified Eagle’s medium (DMEM, Hyclone), supplemented with 10% fetal bovine serum (Corning) and 1% penicillin and streptomycin solution (Corning), at 37°C containing 5% CO_2_.

### Knockdown of ENO1 in T24 and 5637 cells

The shRNA sequence targeting human ENO1 was inserted into pLL3.7 vector between *Hpa*I and *Xho*I site. The shRNA sequences were as follows: ENO1-1 forward: 5′-TCGTGAACGAGAAGTCCTGCAATTCAAGAGATTGCAGGACTTCTCGTTCACGTTTTTTC-3′, ENO1-1 reverse: 5′-TCGAGAAAAAACGTGAACGAGAAGTCCTGCAATCTCTTGAATTGCAGGACTTCTCGTTCACGA-3′; ENO1-2 forward: 5′-TCCACTGTTGAGGTTGATCTCTTTCAAGAGAAGAGATCAACCTCAACAGTGGTTTTTTC-3′, ENO1–2 reverse: 5′-TCGAGAAAAAACCACTGTTGAGGTTGATCTCTTCTCTTGAAAGAGATCAACCTCAACAGTGGA-3′. CTNNB forward: 5′-TGCATAACCTTTCCCATCATCGTTCAAGAGACGATGATGGGAAAGGTTATGCTTTTTTC-3′, CTNNB reverse: 5′-TCGAGAAAAAAGCATAACCTTTCCCATCATCGTCTCTTGAACGATGATGGGAAAGGTTATGCA-3′. pLL3.7-shCtrl vector or pLL3.7-shENO1 vector and packaging vectors (VSVG, REV and pMDL) were co-transfected into 293T cells. 72 h later, culture medium collected from 293T cells was filtered through a 0.45 μm filter and then used to infect T24 and 5637 cells for three times (48 h per time).

### ENO1 or β-catenin over-expression in bladder cancer cells

ENO1 was over-expressed in T24 and 5637 cells using pCDH lentivirus system. Coding sequence (CDS) of *ENO1* or *CTNNB* was synthesized by Generay Biotech Company (Shanghai, China). The cDNA was cloned into *Eco*RI and *Bam*HI sites of pCDH vector. pCDH-Ctrl vector or pCDH-ENO1 vector and packaging vectors (PSPAX2 and PDM2G) were co-transfected into 293T cells. 72 h after transfection, supernatants containing virus were collected and filtered with a 0.45 μm filter. Then the lentivirus was used to infect T24 and 5637 cells for three times (48 h per time). The infection efficiency was evaluated by qRT-PCR and Western blot assays.

### RNA isolation and quantitative real-time PCR

Total RNA was isolated from indicated cells using TRIzol reagent (Invitrogen, U.S.A.). 0.8 μg of total RNA was reverse-transcribed into cDNA using ReverTra Ace® qPCR RT Master Mix (TOYOBO, Japan). Quantitative real-time PCR reaction was performed using TransStart Green qPCR SuperMix (TransGen Biotech, Beijing, China) on a Bio-rad IQ 5 machine. The PCR primer sequences were as follow: ENO1 forward, 5′-GCCTCCTGCTCAAAGTCAAC-3′, and reverse, 5′-AACGATGAGACACCATGACG-3′; cyclin D1 forward, 5′-CAATGACCCCGCACGATTTC-3′, and reverse, 5′-CATGGAGGGCGGATTGGAA-3′; cyclin E1 forward, 5′-GCCAGCCTTGGGACAATAATG-3′, and reverse, 5′-CTTGCACGTTGAGTTTGGGT-3′; caspase 3 forward, 5′-GAAATTGTGGAATTGATGCGTGA-3′, and reverse, 5′-CTACAACGATCCCCTCTGAAAAA-3′; caspase 9 forward, 5′-CTCAGACCAGAGATTCGCAAAC-3′, and reverse, 5′-GCATTTCCCCTCAAACTCTCAA-3′; GAPDH forward: 5′-TGACTTCAACAGCGACACCCA-3′, and reverse: 5′-CACCCTGTTGCTGTAGCCAAA-3′. GAPDH serves as internal control. The expression of target gene = 2^(−(ct(Target)-ct(GAPDH))^. Relative expression was normalized to normal or control group.

### Western blot

Total protein was extracted from indicated cells or tumor tissues using lysis buffer (2% SDS, 100 mM DTT, 10 mM Tris (pH 6.8) and 10% glycerol). Tumor tissues in lysis buffer were sonicated at a frequency of 70 Hz for 180 s. Then they were centrifuged at 12000 rpm for 5 min and boiled at 98°C for 10 min. The protein was separated on a 12% or 15% SDS-PAGE gel and transferred to polyvinylidenefluoride membrane (PVDF; Millipore, U.S.A.). The membranes were blocked with 5% silk milk at room temperature for 1 h and incubated with primary antibodies at 4°C overnight. Antibody against ENO1 (ab85086, 1:1000), cleaved caspase 3 (ab2303) and 9 (ab2324) was from Abcam (U.K.). Antibodies against cyclin D1 (sc-8396, 1:500), E1 (sc-377100, 1:500), p21 (cst2947, 1:1000), p27 (cst3686, 1:1000), caspase 3 (cst9662, 1:1000) and 9 (cst9502, 1:1000) were from Cell Signaling (U.S.A.) and Santa Cruz Biotech (U.S.A.). Antibody against β-catenin (cst8480, 1:1000) was from Cell Signaling (U.S.A.). GAPDH antibody was from ABclonal (AC033, 1:3000) (U.S.A.). All the secondary antibodies (sc-2537 and sc-2005, 1:5000) were from Santa Cruz Biotech (U.S.A.).

### Colony formation assay

A total of 1000 SV-HUC-1, T24 and 5637 cells were seeded in six-well plates. 6 or 10 days later, SV-HUC-1, T24 and 5637 cells were washed respectively with PBS. Then these colonies were fixed with methanol for 15 min and incubated with 0.1% crystal violet for 15 min. Cell colonies were captured with a Nikon camera.

### CCK assay

A total of 2500 indicated SV-HUC-1, T24 and 5637 cells were seeded into 96-well plates, which contained 200 μl of culture medium. 1, 2, 3, 4 and 5 days later, 20 μl of CCK8 solution (YEASEN, Shanghai, China) was added into each well. The plates were incubated at 37°C for 3 h and then the OD value was measured at 450 nm. The OD value of indicated time was normalized to the OD value of day 1.

### Statistical analyses

All the statistical data were represented as mean ± SEM of at least three independent repeats. Unpaired Student’s *t* test was used to analyze the difference between two groups for normally distributed continuous variables. Two-way ANOVA was used for two variables. Difference was considered significant when *P* <0.05.

## Results

### ENO1 is over-expressed in bladder cancer tissues and cells

**Figure 1:**
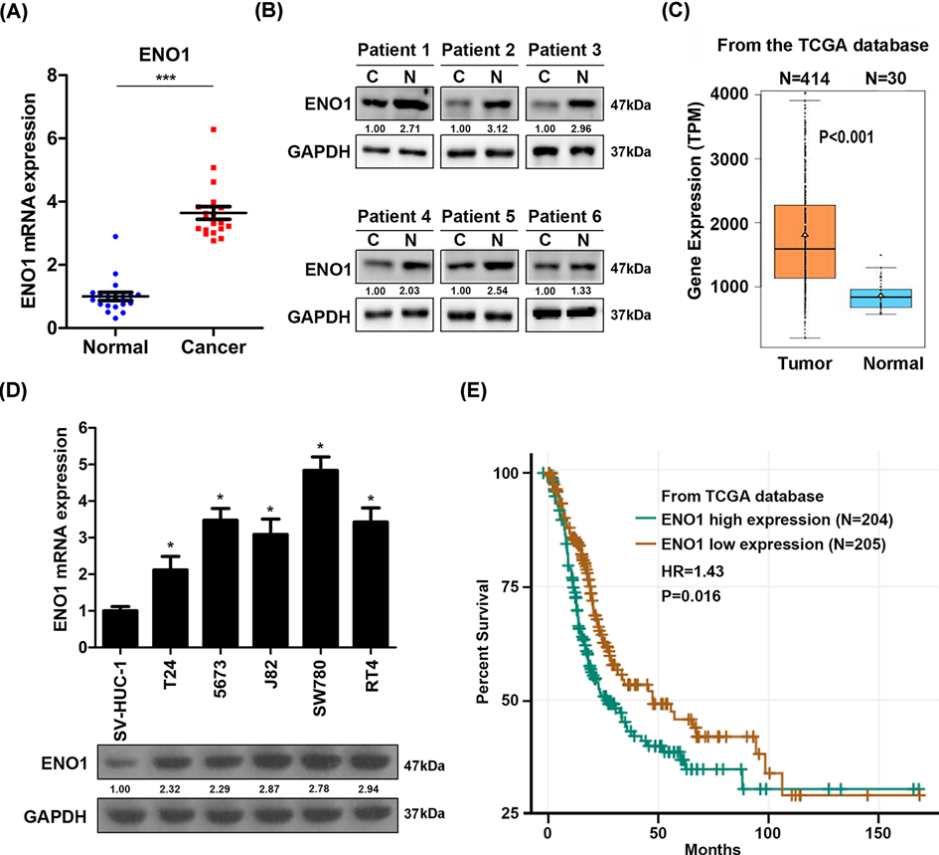
ENO1 expression is increased in BC tissues and cells (**A**) qRT-PCR analysis of ENO1 in BC and normal tissues. ENO1 expression in BC tissues was normalized to ENO1 expression in normal tissues. *n* = 19 in normal, *n* = 19 in cancer. ***P<0.001. (**B**) Western blot analysis of ENO1 in bladder cancer and adjacent normal tissues. (**C**) mRNA level of ENO1 in bladder cancer and normal tissues that were analyzed from TCGA database. TPM, transcripts per million. *P*<0.001. (**D**) qRT-PCR and Western blot analysis of ENO1 in bladder epithelial cells SV-HUC-1 and in BC cells T24, 5637, J82, SW780 and RT4. ENO1 expression in T24, 5637, J82, SW780 and RT4 was normalized to ENO1 expression in SV-HUC-1 cells. *P<0.05, **P<0.01. (**E**) The overall survival of bladder cancer patients who were divided into ENO1 high- and low-expression groups that were analyzed from TCGA database. *n* = 204 in low-expression group, *n* = 205 in high-expression group. *P*=0.016.

To investigate the clinical relevance of ENO1 in BC, we collected BC and the normal tissues. qRT-PCR or Western blot results showed that ENO1 mRNA and protein level was higher in BC tissues compared with the non-paired or paired normal bladder tissues (Figure 1A,B). To confirm our results, we analyzed the transcriptional level of ENO1 in BC and separate normal tissues from the TCGA database. We found that ENO1 mRNA expression was higher in BC tissues as compared with the normal tissues (Figure 1C). Furthermore, the mRNA level of ENO1 was also determined in various BC cells. The BC cells, including T24, 5637, J82, SW780 and RT4, had increased ENO1 mRNA expression comparing to bladder epithelial cells SV-HUC-1 (Figure 1D). In addition, the correlation between ENO1 expression and bladder cancer survival was analyzed based on the TCGA database. The results showed that the patients with ENO1 high expression exhibited poor survival than those with ENO1 low expression (Figure 1E). Taken together, ENO1 is a promising diagnostic biomarker for BC.

### ENO1 knockdown suppresses the BC cell proliferation and growth

**Figure 2:**
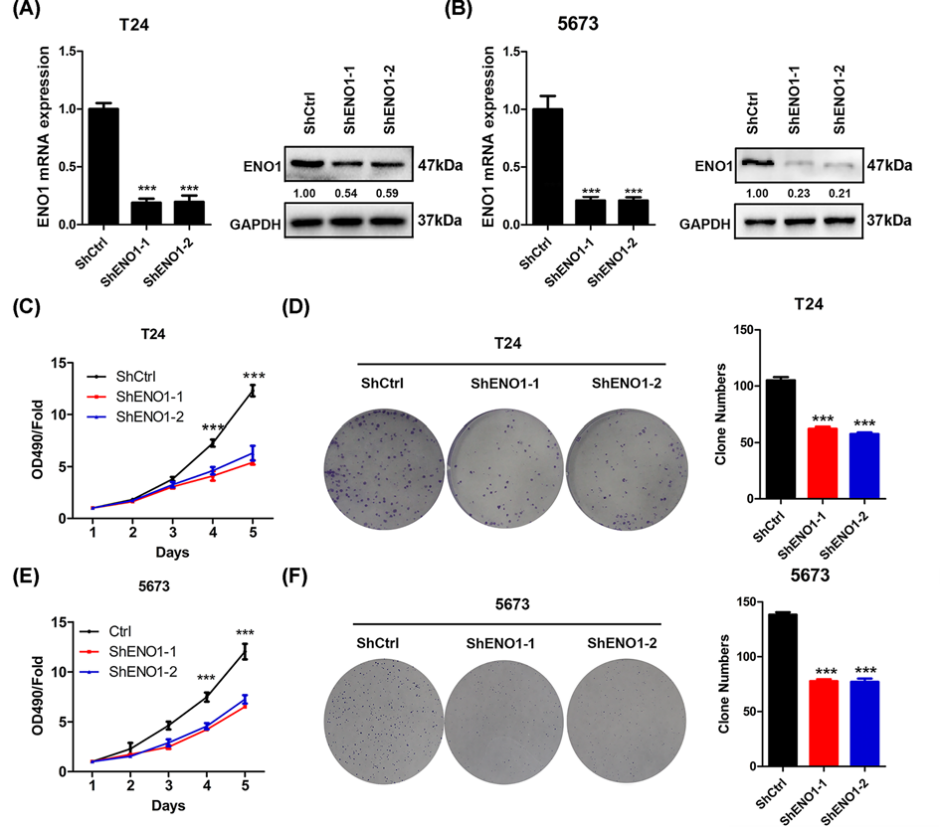
ENO1 knockdown inhibits the proliferation and colony formation of bladder cancer cells (**A**) T24 cells were infected with shCtrl, shENO1-1 and shENO1-2 lentivirus for three times and then were subjected to qRT-PCR and Western blot analysis of ENO1. ***P<0.001. (**B**) 5637 cells were infected with shCtrl, shENO1-1 and shENO1-2 lentivirus for three times and then were subjected to qRT-PCR and Western blot analysis of ENO1. ***P<0.001. (**C**) shCtrl, shENO1-1 and shENO1-2 T24 cells were subjected to CCK analysis of proliferation. ***P<0.001. (**D**) Cells described in (C) were subjected to colony formation analysis. Left, representative images. Right, quantification results. ***P<0.001. (**E**) shCtrl, shENO1-1 and shENO1-2 5637 cells were subjected to CCK analysis. ***P<0.001. (**F**) Cells described in (E) were subjected to colony formation analysis. Left, representative images. Right, quantification results. ***P<0.001.

Since ENO1 expression was increased in BC tissues and cells, we explored whether ENO1 participated in BC progression. We knocked down ENO1 in T24 and 5637 cells and then analyzed the cell proliferation and growth. qRT-PCR and Western blot results showed that ENO1 was efficiently silenced in shENO1-1 and shENO1-2 T24 and 5637 cells (Figure 2A,B). Silencing of ENO1 suppressed the proliferation and colony formation of T24 cells (Figure 2C,D). Consistent results were observed in shENO1-1 and shENO1-2 5637 cells (Figure 2E,F).

### ENO1 over-expression promotes the BC cell proliferation and growth

**Figure 3:**
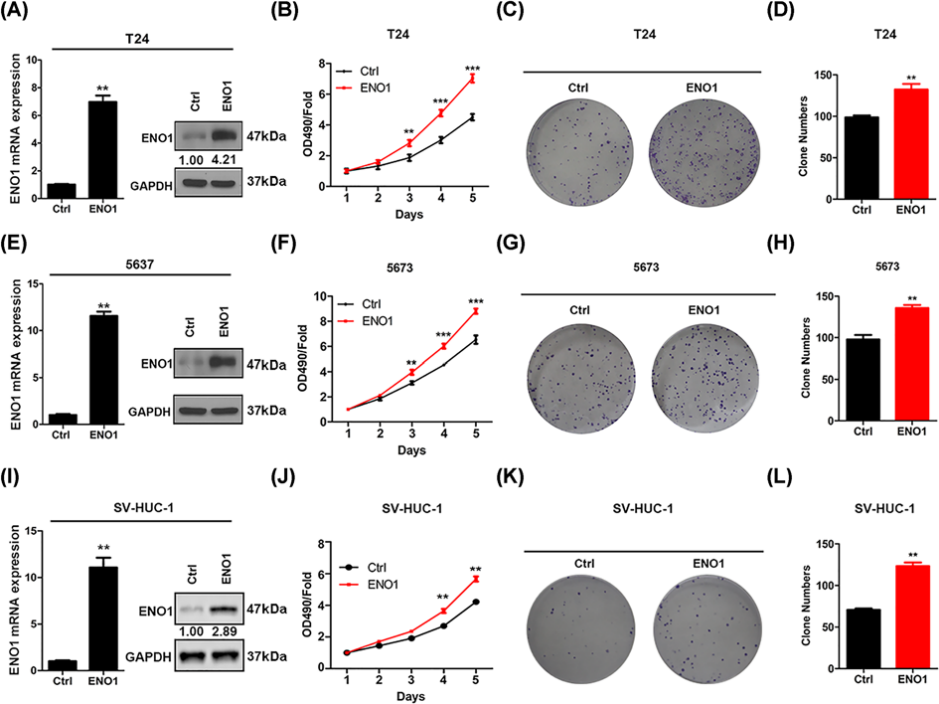
ENO1 knockdown inhibits the proliferation and colony formation of bladder cancer cells (**A**) T24 cells were infected with Ctrl and ENO1 over-expression lentivirus and then were subjected to qRT-PCR and Western blot analysis of ENO1. ***P<0.001. (**B**) 5637 cells were infected with Ctrl and ENO1 over-expression lentivirus and then were subjected to qRT-PCR and Western blot analysis of ENO1. ***P<0.001. (**C**) SV-HUC-1 cells were infected with Ctrl and ENO1 over-expression lentivirus and then were subjected to qRT-PCR and Western blot analysis of ENO1. ***P<0.001. (**D**–**F**) Ctrl and ENO1 over-expressed T24 cells were subjected to CCK analysis of proliferation (D) and colony formation assay (E and F). **P<0.01, ***P<0.001. (**G**–**I**) Ctrl and ENO1 over-expressed 5637 cells were subjected to CCK analysis of proliferation (G) and colony formation assay (H and I). **P<0.01, ***P<0.001. (**J**–**L**) Ctrl and ENO1 over-expressed SV-HUC-1 cells were subjected to CCK analysis of proliferation (J) and colony formation assay (K and L). **P<0.01.

To confirm our findings, we over-expressed ENO1 in T24 and 5637 cells and checked the effect of ENO1 ectopic expression on cell proliferation and growth (Figure 3). qRT-PCR and Western blot assays indicated that ENO1 was over-expressed in T24 and 5637 cells (Figure 3A,B). ENO1 ectopic expression led to enhanced cell proliferation and colony formation of both T24 and 5637 cells (Figure 3D,I). The results showed that ENO1 over-expression promoted the growth of T24 and 5637 cells. We also over-expressed ENO1 in SV-HUC-1 cells (Figure 3C). The results showed that ENO1 ectopic expression enhanced the proliferation and growth of SV-HUC-1 cells (Figure 3J,L). Therefore, ENO1 is essential to BC cell proliferation and growth.

### ENO1 regulates apoptosis, cell cycle and activates β-catenin

**Figure 4:**
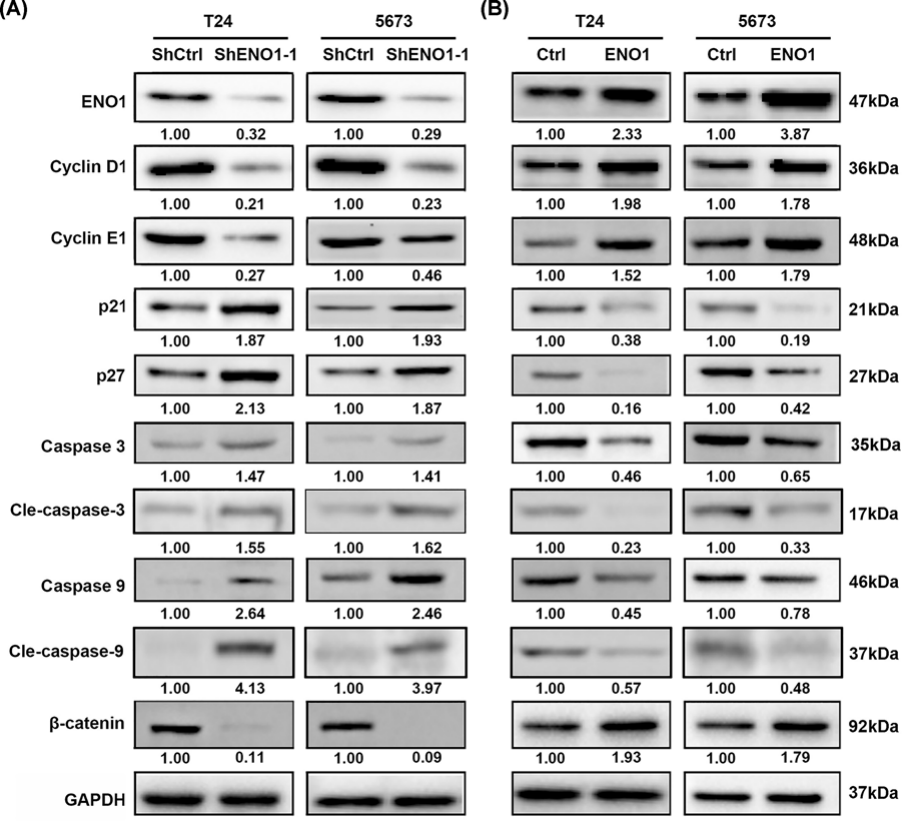
ENO1 regulates cell cycle, apoptosis and enhances β-catenin signaling pathway in bladder cancer cells (**A**) Western blot analysis of ENO1, cyclin D1, cyclin E1, p21, p27, caspase 3, caspase 9 and β-catenin in shCtrl and shENO1-1 T24 and 5637 cells. (**B**) Western blot analysis of ENO1, cyclin D1, cyclin E1, p21, p27, caspase 3, cleaved caspase 3, caspase 9, cleaved caspase 9 and β-catenin in Ctrl and ENO1 over-expressed T24 and 5637 cells.

Next, we attempted to explore the molecular mechanisms contributing to the oncogenic role of ENO1 in bladder cancer. Cancer cells always exhibit enhanced cell cycle progression and suppressed apoptosis. We firstly checked the dys-regulated genes which are associated with cell cycle and apoptosis. Western blot results showed that cyclin D1 and E1 expression were suppressed by ENO1 knockdown, while they were potentiated by ENO1 over-expression (Figure 4A,B). p21 and p27, the repressor of cell cycle progression, were negatively regulated by ENO1 (Figure 4A,B). Furthermore, the apoptosis-related genes, caspase 3, caspase 9 and their active forms were up-regulated and down-regulated in ENO1 silencing and over-expressed BC cells, respectively (Figure 4A,B). β-catenin is frequently activated in different cancers, including bladder cancer. Cyclin D1 has been reported as the downstream effector of β-catenin. We showed that ENO1 positively regulated β-catenin in both T24 and 5637 cells (Figure 4A,B). These results suggest that ENO1 regulates cell cycle, apoptosis and activates β-catenin signaling pathway in bladder cancer cells.

### ENO1 activation of β-catenin is critical for the bladder cancer cell proliferation

**Figure 5:**
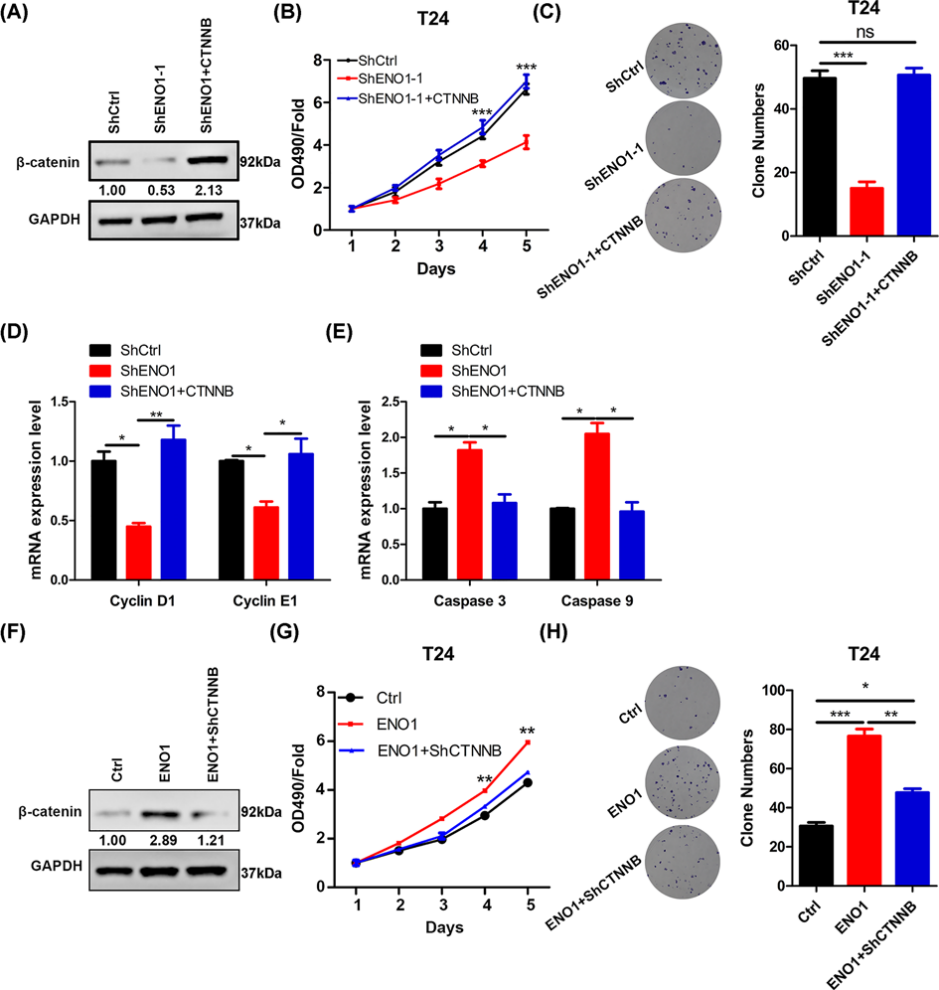
ENO1 up-regulation of β-catenin promotes the bladder cancer cell proliferation and colony formation (**A**) β-catenin was over-expressed in shENO1-1 T24 cells and the cells (shCtrl, shENO1-1, shENO1-1+CTNNB) were subjected to Western blot analysis of β-catenin. (**B**) shCtrl, shENO1-1 and shENO1-1 with over-expressed β-catenin T24 cells were subjected to CCK analysis of proliferation. ***P<0.001. (**C**) The cells described in B were subjected to colony formation analysis. Left, representative images. Right, quantification results. ***P<0.001. (**D** and **E**) qRT-PCR results of cyclin D1 and E1 (D), caspase 3 and 9 (E) in the cells described in B. *P<0.05, **P<0.01. (**F**) β-catenin was silenced in ENO1 over-expressed T24 cells and the cells (Ctrl, ENO1, ENO1+shCTNNB) were subjected to Western blot analysis of β-catenin. (**G**) Ctrl, ENO1 and ENO1+shCTNNB T24 cells were subjected to CCK analysis of proliferation. **P<0.01. (**H**) The cells described in B were subjected to colony formation analysis. Left, representative images. Right, quantification results. *P<0.05, **P<0.01, ***P<0.001.

To investigate the involvement of β-catenin on bladder cancer, we over-expressed β-catenin in ENO1 silenced T24 cells (Figure 5). We found that β-catenin ectopic expression promoted the proliferation and colony growth of T24 cells with silenced ENO1 (Figure 5A,C). Furthermore, cyclin D1 and cyclin E1, which were repressed by ENO1 knockdown, were re-enhanced by β-catenin over-expression (Figure 5D). Opposite results were observed in qRT-PCR analysis of caspase 3 and caspase 9 (Figure 5E). In addition, we silenced β-catenin in ENO1 over-expressed T24 cells and found that β-catenin knockdown suppressed the proliferation and growth of T24 cells (Figure 5F,H). Thus, ENO1 up-regulation of β-catenin is critical for the bladder cancer cell proliferation.

## Discussion

In this study, we found that ENO1 was up-regulated in BC tissues and cells. ENO1 over-expression promoted BC cell proliferation and growth. Opposite results were observed in ENO1 silenced BC cells. Cell cycle and apoptosis-associated genes were dys-regulated in ENO1 over-expressed and silenced BC cells. Our study demonstrated that ENO1 functioned as an oncogene in BC.

In the past decades, numerous studies have demonstrated that aerobic glycolysis, the ‘Warburg effect’, is a hallmark of cancer [29]. Even though increasing evidences have shown that the critical glycolytic enzymes, including HK2, PKM2 and LDHA, play important roles in cancer development, the drugs against these enzymes are far from clinical application [11]. Therefore, further studies should be performed to identify other glycolytic enzymes in cancer progression. Recently, the glycolytic enzyme ENO1 has attracted the attention of oncologists because it is dys-regulated in various cancer types, including gastric cancer, HCC, glioma and pancreatic cancer [14,30–32]. However, it remains unknown whether ENO1 serves as a tumor suppressor, oncogene or neither in BC. In this study, we focused on the involvement of ENO1 in BC development. We found that the protein and mRNA level of ENO1 was up-regulated in BC samples. Different BC cells showed relatively higher mRNA expression of ENO1 than the bladder epithelial cells SV-HUC-1. *In vitro*, ectopic expression of ENO1 accelerated the proliferation and colony formation of T24 and 5637 BC cells, which had moderate ENO1 expression. Opposite results were observed in ENO1 silenced T24 and 5637 cells. The proliferative potential of SV-HUC-1 was also enhanced by ENO1 over-expression. These results suggested that ENO1 functioned as an oncogene in BC. We also attempted to identify down-stream effectors that are regulated by ENO1. We showed that ENO1 positively regulated β-catenin expression in both T24 and 5637 cells.

Due to alterations of any components in this pathway, Wnt/β-catenin signaling pathway is aberrantly activated in various cancers. β-catenin can also be activated or stabilized by other factors to promote cancer development. For example, long noncoding RNA pancEts-1 accelerates neuroblastoma progression via hnRNPK-mediated stabilization of β-catenin [33]. Cytoplasmic hPCL3s, which is highly expressed in HCC samples, promotes HCC metastasis through activation of β-catenin/IL6 pathway [34]. After stabilizing or activating by Wnt signals or other factors, β-catenin enters the nucleus and elicits its transcriptional activity. The most initially identified targets for β-catenin are c-myc and cyclin D1, which act as oncogenic roles in cancer development. Additionally, Deptor, a suppressor of mTOR, has been demonstrated as a novel target of Wnt/β-catenin/c-Myc signaling pathway and contributes to the growth of colorectal cancer cell [35]. Here, we showed that the expression of β-catenin as well as its targets cyclin D1 was increased by ENO1 over-expression. Conversely, ENO1 silencing resulted in down-regulation of β-catenin and cyclin D1. These results suggested that ENO1 activated β-catenin signaling. However, this did not imply that β-catenin activation participates in BC cell proliferation triggered by ENO1. We thus over-expressed β-catenin in BC cells with silenced ENO1. Interestingly, ectopic expression of β-catenin rescued the cell proliferation of ENO1 knockdown T24 cells. In contrast, knockdown of β-catenin reduced the viability of ENO1 over-expressed T24 cells. Our results indicated that β-catenin signaling is critical for the oncogenic role of ENO1 in BC cell proliferation.

Even though we demonstrated that ENO1/β-catenin cascade played an important role in BC, there were still some limitations in our study. How ENO1 regulated β-catenin expression remained unclear. A previous study found that ENO1 was positively regulated by β-catenin in murine alveolar epithelial cells [36]. In turn, another study showed that β-catenin served as a downstream effecter of ENO1 in glioma [37]. They also identified that PI3K/AKT signaling pathway was suppressed by ENO1 knockdown. In addition, inhibition of PI3K by LY294002 reduced the epithelial–mesenchymal transition (EMT) and the expression of cyclin D1. These results suggest that ENO1 may regulate β-catenin through PI3K/AKT signaling. Consistently, we provided the evidence that ENO1 potentiated β-catenin expression in BC cells. However, further studies should be conducted to illustrate the precise molecular mechanisms between ENO1 and β-catenin.

In summary, we demonstrated ENO1 as a potential oncogene in BC. Up-regulated ENO1 in BC tissues promoted the proliferation and colony formation of BC cells. Mechanistically, activation of β-catenin signaling pathway was essential to the accelerated proliferation of ENO1 over-expressed BC cells. Therefore, ENO1/β-catenin cascade maybe a potential therapeutic target for BC.

## Abbreviations

BC: bladder cancer
ENO1: enolase 1
DMEM: Dulbecco modified Eagle’s medium
HCC: hepatocellular carcinoma
qRT-PCR: quantitative real-time PCR
