# Expression of Concern: Up-regulated ENO1 promotes the bladder cancer cell growth and proliferation via regulating β-catenin

**DOI:** 10.1042/BSR-20190503_EOC

**Published:** 2021-02-16

**Keywords:** β-catenin, apoptosis, bladder cancer, cell cycle, ENO1

The Editorial Office has been made aware of potential issues surrounding the scientific validity of this paper, hence has issued an expression of concern to notify readers whilst the Editorial Office investigates.

