# Correction: Up-regulated ENO1 promotes the bladder cancer cell growth and proliferation via regulating β-catenin

**DOI:** 10.1042/BSR-2019-0503_COR

**Published:** 2021-10-29

**Keywords:** apoptosis, bladder cancer, cell cycle, ENO1

This Correction follows an Expression of Concern relating to this article previously published by Portland Press.

The authors of the original article “Up-regulated ENO1 promotes the bladder cancer cell growth and proliferation via regulating β-catenin” (*Biosci Rep* (2019) **39**(9); https://doi.org/10.1042/BSR20190503) would like to correct the western blots in their figures. The authors state that in their original Figures 1-5, the western blot bands were obscure due to the chemiluminescence machine used. They have repeated the whole experiment on request by the Editorial Board, with a higher resolution setting. The authors declare that the results of their new experiment do not affect the results and conclusions of their original article.

**Figure 1:**
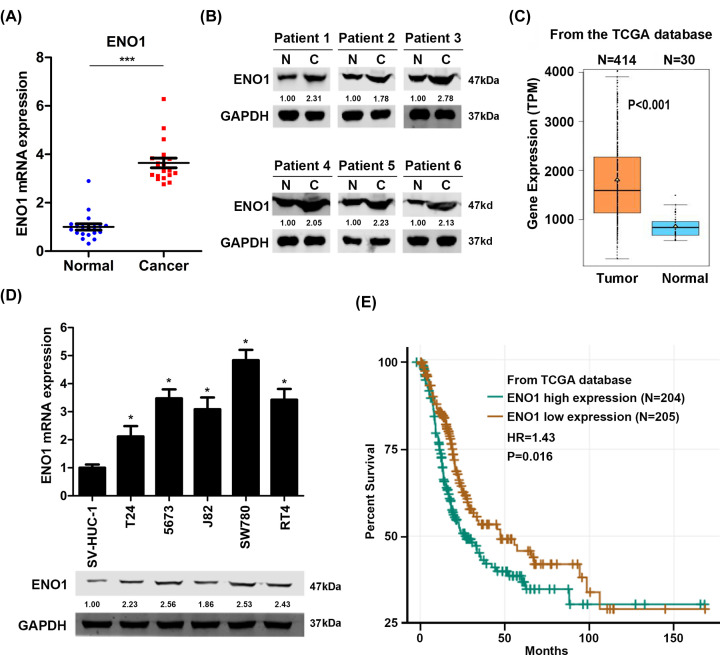
ENO1 expression is increased in BC tissues and cells (**A**) qRT-PCR analysis of ENO1 in BC and normal tissues. ENO1 expression in BC tissues was normalized to ENO1 expression in normal tissues. *n* = 19 in normal, *n* = 19 in cancer. ***P<0.001. (**B**) Western blot analysis of ENO1 in bladder cancer and adjacent normal tissues. (**C**) mRNA level of ENO1 in bladder cancer and normal tissues that were analyzed from TCGA database. TPM, transcripts per million. *P*<0.001. (**D**) qRT-PCR and Western blot analysis of ENO1 in bladder epithelial cells SV-HUC-1 and in BC cells T24, 5637, J82, SW780 and RT4. ENO1 expression in T24, 5637, J82, SW780 and RT4 was normalized to ENO1 expression in SV-HUC-1 cells. *P<0.05, **P<0.01. (**E**) The overall survival of bladder cancer patients who were divided into ENO1 high- and low-expression groups that were analyzed from TCGA database. *n* = 204 in low-expression group, *n* = 205 in high-expression group. *P* = 0.016.

**Figure 2:**
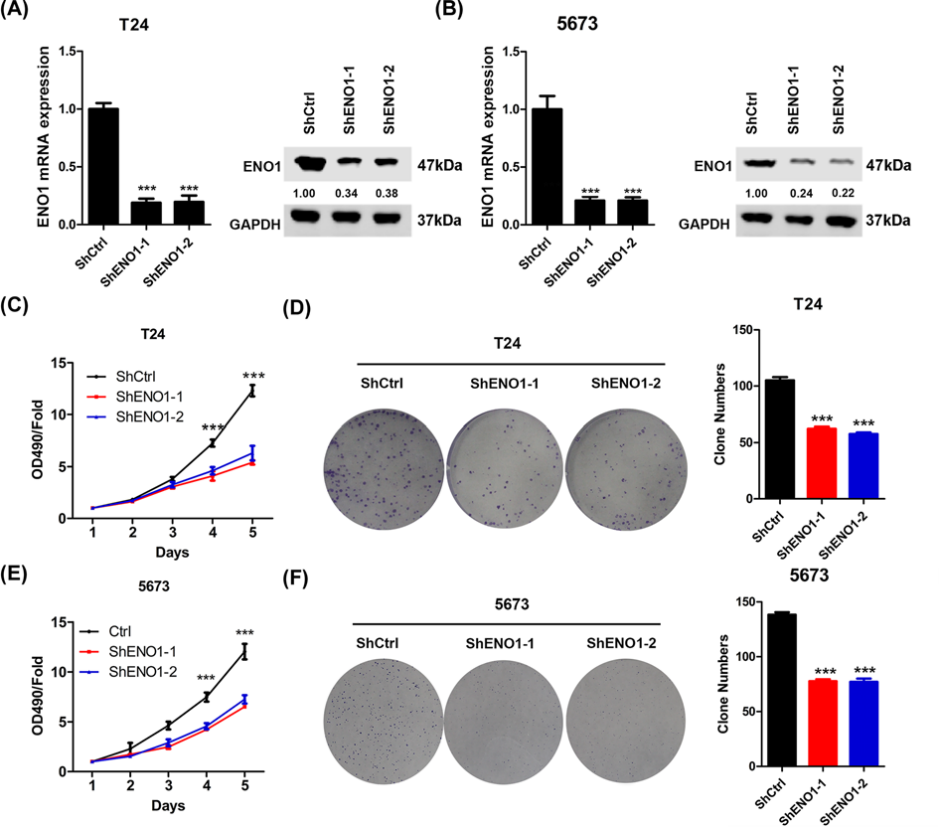
ENO1 knockdown inhibits the proliferation and colony formation of bladder cancer cells (**A**) T24 cells were infected with shCtrl, shENO1-1 and shENO1-2 lentivirus for three times and then were subjected to qRT-PCR and Western blot analysis of ENO1. ***P<0.001. (**B**) 5637 cells were infected with shCtrl, shENO1-1 and shENO1-2 lentivirus for three times and then were subjected to qRT-PCR and Western blot analysis of ENO1. ***P<0.001. (**C**) shCtrl, shENO1-1 and shENO1-2 T24 cells were subjected to CCK analysis of proliferation. ***P<0.001. (**D**) Cells described in (C) were subjected to colony formation analysis. Left, representative images. Right, quantification results. ***P<0.001. (**E**) shCtrl, shENO1-1 and shENO1-2 5637 cells were subjected to CCK analysis. ***P<0.001. (**F**) Cells described in (E) were subjected to colony formation analysis. Left, representative images. Right, quantification results. ***P<0.001.

**Figure 3:**
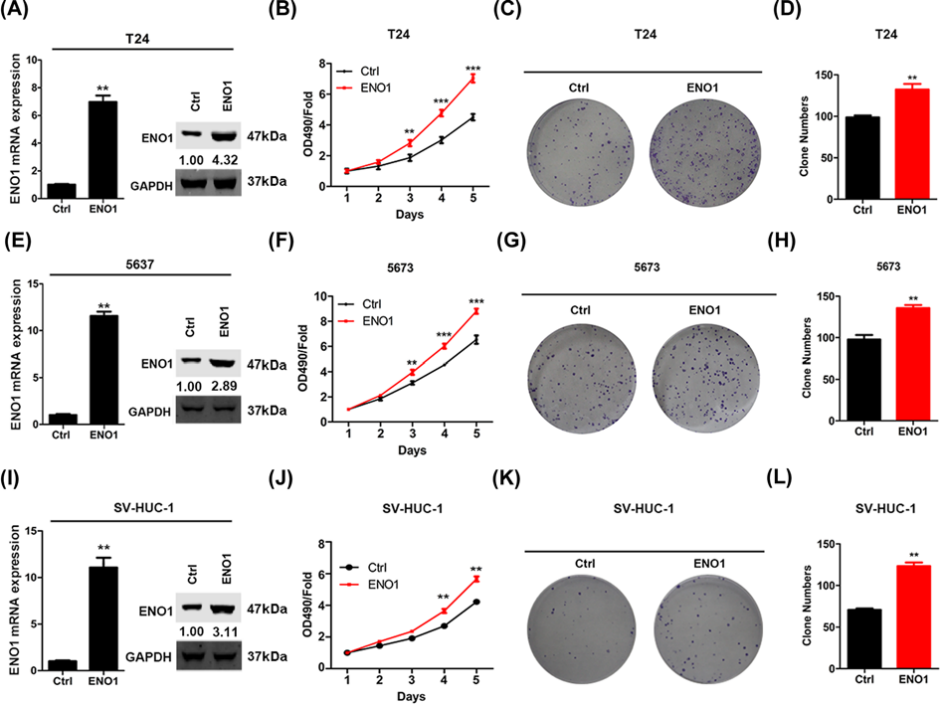
ENO1 knockdown inhibits the proliferation and colony formation of bladder cancer cells (**A**) T24 cells were infected with Ctrl and ENO1 over-expression lentivirus and then were subjected to qRT-PCR and Western blot analysis of ENO1. ***P<0.001. (**B**) 5637 cells were infected with Ctrl and ENO1 over-expression lentivirus and then were subjected to qRT-PCR and Western blot analysis of ENO1. ***P<0.001. (**C**) SV-HUC-1 cells were infected with Ctrl and ENO1 over-expression lentivirus and then were subjected to qRT-PCR and Western blot analysis of ENO1. ***P<0.001. (**D**–**F**) Ctrl and ENO1 over-expressed T24 cells were subjected to CCK analysis of proliferation (D) and colony formation assay (E and F). **P<0.01, ***P<0.001. (**G**–**I**) Ctrl and ENO1 over-expressed 5637 cells were subjected to CCK analysis of proliferation (G) and colony formation assay (H and I). **P<0.01, ***P<0.001. (**J**–**L**) Ctrl and ENO1 over-expressed SV-HUC-1 cells were subjected to CCK analysis of proliferation (J) and colony formation assay (K and L). **P<0.01.

**Figure 4:**
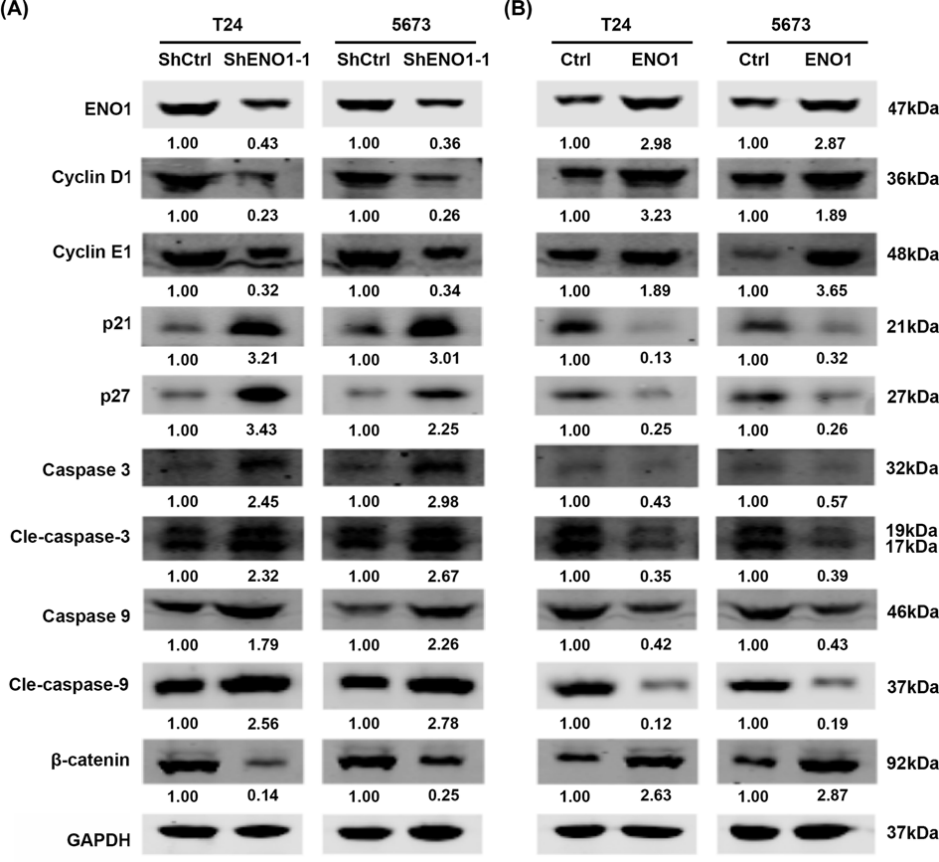
ENO1 regulates cell cycle, apoptosis and enhances β-catenin signaling pathway in bladder cancer cells (**A**) Western blot analysis of ENO1, cyclin D1, cyclin E1, p21, p27, caspase 3, caspase 9 and β-catenin in shCtrl and shENO1-1 T24 and 5637 cells. (**B**) Western blot analysis of ENO1, cyclin D1, cyclin E1, p21, p27, caspase 3, cleaved caspase 3, caspase 9, cleaved caspase 9 and β-catenin in Ctrl and ENO1 over-expressed T24 and 5637 cells.

**Figure 5:**
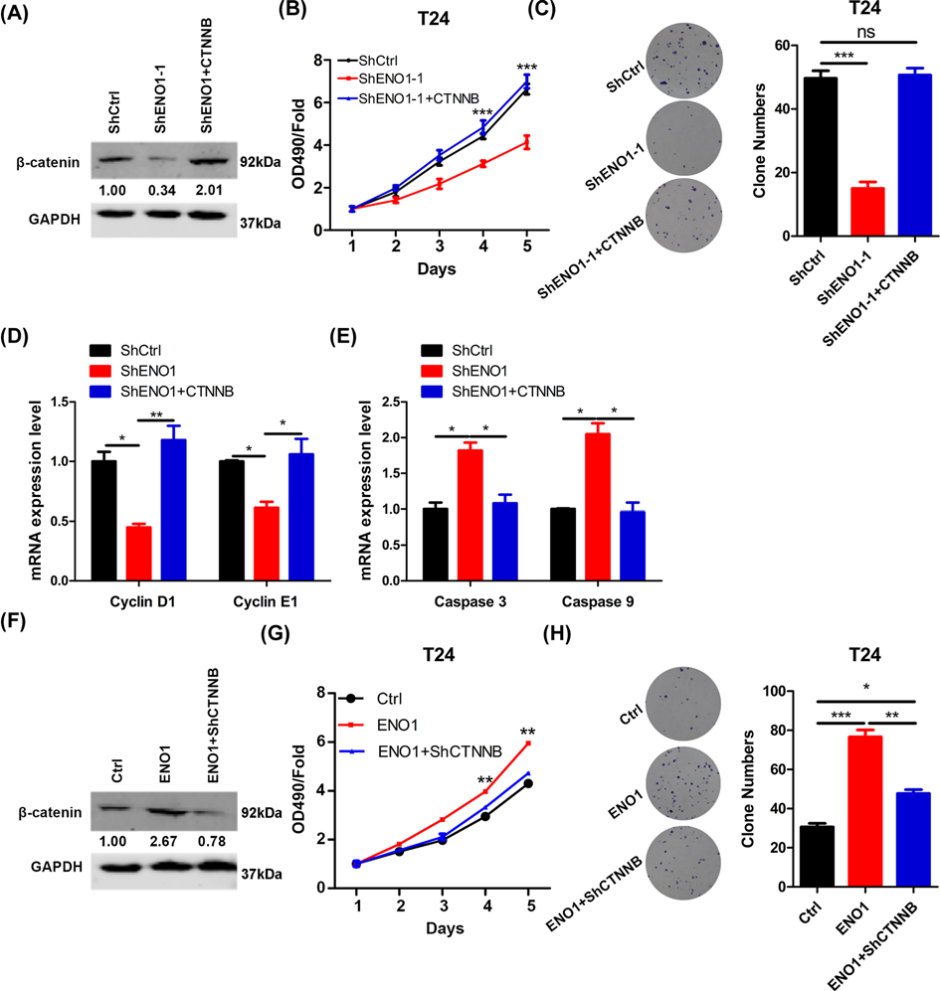
ENO1 up-regulation of β-catenin promotes the bladder cancer cell proliferation and colony formation (**A**) β-catenin was over-expressed in shENO1-1 T24 cells and the cells (shCtrl, shENO1-1, shENO1-1+CTNNB) were subjected to Western blot analysis of β-catenin. (**B**) shCtrl, shENO1-1 and shENO1-1 with over-expressed β-catenin T24 cells were subjected to CCK analysis of proliferation. ***P<0.001. (**C**) The cells described in B were subjected to colony formation analysis. Left, representative images. Right, quantification results. ***P<0.001. (**D** and **E**) qRT-PCR results of cyclin D1 and E1 (D), caspase 3 and 9 (E) in the cells described in B. *P<0.05, **P<0.01. (**F**) β-catenin was silenced in ENO1 over-expressed T24 cells and the cells (Ctrl, ENO1, ENO1+shCTNNB) were subjected to Western blot analysis of β-catenin. (**G**) Ctrl, ENO1 and ENO1+shCTNNB T24 cells were subjected to CCK analysis of proliferation. **P<0.01. (**H**) The cells described in B were subjected to colony formation analysis. Left, representative images. Right, quantification results. *P<0.05, **P<0.01, ***P<0.001.

